# Expression Level and Subcellular Localization of Heme Oxygenase-1 Modulates Its Cytoprotective Properties in Response to Lung Injury: A Mouse Model

**DOI:** 10.1371/journal.pone.0090936

**Published:** 2014-03-05

**Authors:** Fumihiko Namba, Hayato Go, Jennifer A. Murphy, Ping La, Guang Yang, Shaon Sengupta, Amal P. Fernando, Mekdes Yohannes, Chhanda Biswas, Suzanne L. Wehrli, Phyllis A. Dennery

**Affiliations:** 1 Division of Neonatology, Children’s Hospital of Philadelphia, Philadelphia, Pennsylvania, United States of America; 2 Department of Pediatrics, University of Pennsylvania, Philadelphia, Pennsylvania, United States of America; 3 Small Animal Core, Children’s Hospital of Philadelphia, Philadelphia, Pennsylvania, United States of America; University of Pecs Medical School, Hungary

## Abstract

Premature infants exposed to hyperoxia suffer acute and long-term pulmonary consequences. Nevertheless, neonates survive hyperoxia better than adults. The factors contributing to neonatal hyperoxic tolerance are not fully elucidated. In contrast to adults, heme oxygenase (HO)-1, an endoplasmic reticulum (ER)-anchored protein, is abundant in the neonatal lung but is not inducible in response to hyperoxia. The latter may be important, because very high levels of HO-1 overexpression are associated with significant oxygen cytotoxicity in vitro. Also, in contrast to adults, HO-1 localizes to the nucleus in neonatal mice exposed to hyperoxia. To understand the mechanisms by which HO-1 expression levels and subcellular localization contribute to hyperoxic tolerance in neonates, lung-specific transgenic mice expressing high or low levels of full-length HO-1 (cytoplasmic, HO-1-FL(H) or HO-1-FL(L)) or C-terminally truncated HO-1 (nuclear, Nuc-HO-1-TR) were generated. In HO-1-FL(L), the lungs had a normal alveolar appearance and lesser oxidative damage after hyperoxic exposure. In contrast, in HO-1-FL(H), alveolar wall thickness with type II cell hyperproliferation was observed as well worsened pulmonary function and evidence of abnormal lung cell hyperproliferation in recovery from hyperoxia. In Nuc-HO-1-TR, the lungs had increased DNA oxidative damage, increased poly (ADP-ribose) polymerase (PARP) protein expression, and reduced poly (ADP-ribose) (PAR) hydrolysis as well as reduced pulmonary function in recovery from hyperoxia. These data indicate that low cytoplasmic HO-1 levels protect against hyperoxia-induced lung injury by attenuating oxidative stress, whereas high cytoplasmic HO-1 levels worsen lung injury by increasing proliferation and decreasing apoptosis of alveolar type II cells. Enhanced lung nuclear HO-1 levels impaired recovery from hyperoxic lung injury by disabling PAR-dependent regulation of DNA repair. Lastly both high cytoplasmic and nuclear expression of HO-1 predisposed to long-term abnormal lung cellular proliferation. To maximize HO-1 cytoprotective effects, therapeutic strategies must account for the specific effects of its subcellular localization and expression levels.

## Introduction

Premature neonates with altered lung function are exposed to hyperoxia to maintain adequate oxygenation but they may develop bronchopulmonary dysplasia, a pulmonary disease with long-term sequelae including neurodevelopmental delay [1]–[3]. Interestingly, humans, in particular neonates, have developed some adaptive mechanisms to mitigate oxidative stress. Although prolonged hyperoxic exposure causes injury to the neonatal lung, neonatal rodents are more tolerant to hyperoxia than their adult counterparts [4]. Some have implicated increased antioxidant enzyme activity and a reduced superoxide-generating capacity in the neonatal lung [5]–[7]. Nevertheless, these observations do not fully explain the enhanced hyperoxic tolerance in neonates. One molecule with antioxidant properties, HO-1, the rate-limiting enzyme in the degradation of heme, is robustly inducible in oxidative stress such as hyperoxia in adults [8] but differentially regulated in neonatal rodents as compared to the adult [9]. Despite increased HO-1 expression in the perinatal period, there was no difference in lung HO-1 mRNA levels in newborn rats (<12 hours old) exposed to hyperoxia, for 3 days compared to air exposed controls, in contrast with adult models.

Whereas HO-1 knockout mice which have a shorter life span, reduced stress defenses, and disrupted postnatal lung development [10], [11], lung-specific neonatal HO-1 transgenic mice demonstrated vasculoprotective effects in hyperoxia [12]. However, very high levels of HO-1 expression were associated with significant oxygen cytotoxicity *in vitro*, suggesting there is a beneficial threshold of HO-1 overexpression [13]. Whether this is the case *in vivo* is not known. The HO-1 protein is anchored to the ER through a transmembrane segment located at the C-terminus [14], with the remainder in the cytoplasm [15]. Nuclear localization of HO-1 has been demonstrated in many situations including in astroglial cells during differentiation [16], in fetal lung cells under hyperoxia [17], and in brown adipocytes [18]. Interestingly, nuclear HO-1 was implicated as a regulator of DNA repair activities important to carcinogenesis [19], [20] and tumor progression [21]. We have shown that HO-1 can be proteolytically cleaved from the ER to allow nuclear translocation with hypoxia [22]. This may serve to upregulate cytoprotective genes against oxidative stress [22]. Nuclear HO-1 is found in higher abundance in the lungs from neonatal mice exposed to hyperoxia compared to similarly exposed adults [23]. Could this translocation of cytoplasmic HO-1 to the nucleus contribute to tolerance against oxidative stress and promote or protect against abnormal cell proliferation and tumorigenesis *in vivo*?

To better understand the impact of HO-1 abundance and subcellular localization on hyperoxic injury and repair, lung-specific HO-1 transgenic mice were developed to express either low or high levels of cytoplasmic HO-1 or enhanced nuclear HO-1.

## Materials and Methods

### HO-1 Transgenic Lines

**Figure 1:**
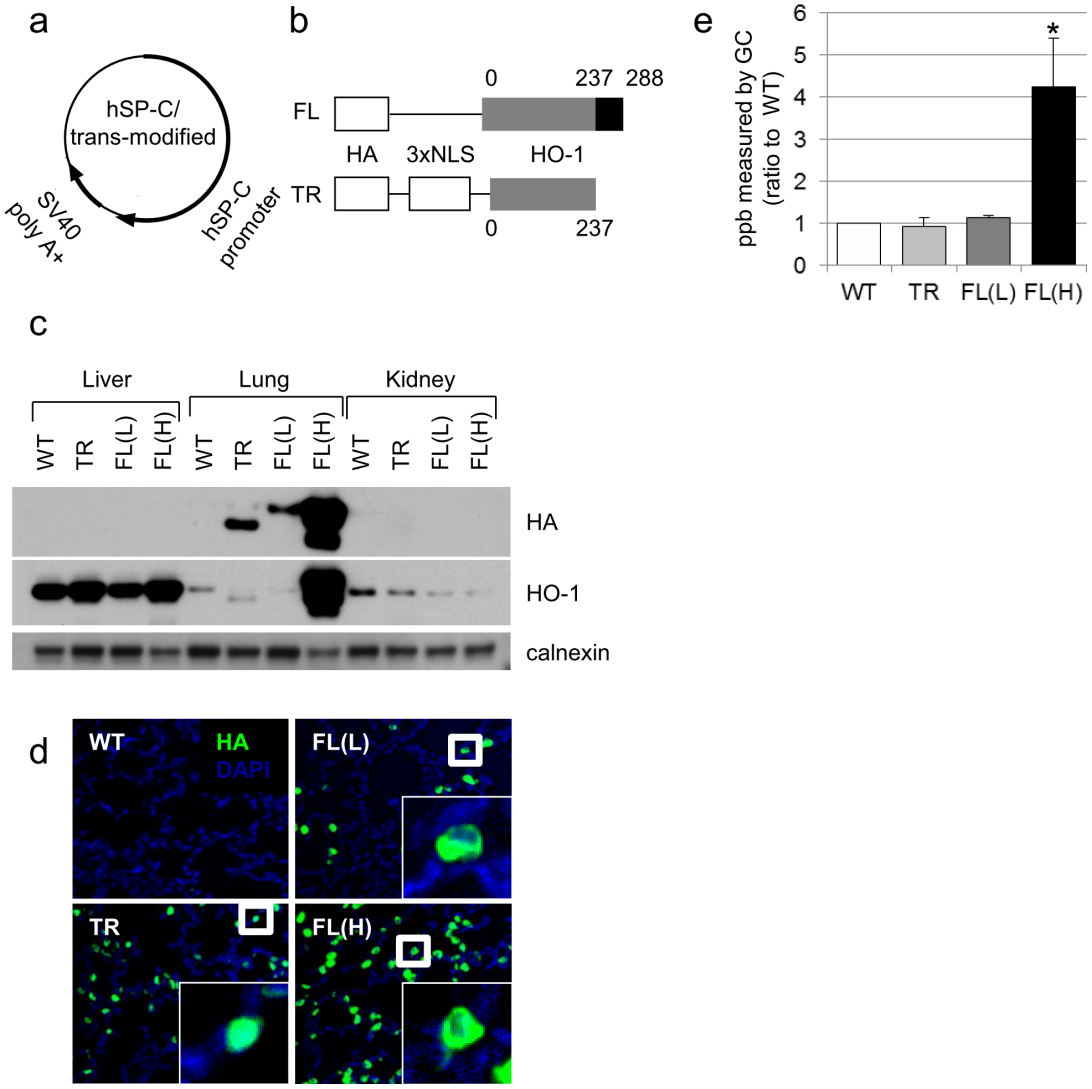
Expression, localization, and HO activity of HA-tagged HO-1 protein in the murine lung. (**a**) Human SP-C/trans-modified vector construct. (**b**) HA-tagged HO-1 cDNA constructs. Numbers denote amino acids in the fragment starting from the N terminus. NLS, nuclear localization sequence. (**c**) Immunoblots showing lung-specific overexpression of high or low levels of HA-tagged HO-1-FL (FL(H) or FL(L)) or Nuc-HO-1-TR (TR). Membranes were re-probed with calnexin antibodies as a loading control. (**d**) HA immunostaining (green) and nuclear DAPI staining (blue) in lung slices from HO-1-FL (FL) and Nuc-HO-1-TR (TR) transgenic mice. (**e**) Total lung HO activity in whole lung homogenates on day 14 of life. Values are the mean ± SEM of 3 separate determinations in each group. *, p<0.05 vs WT.

All procedures and protocols were reviewed and approved by the Children’s Hospital of Philadelphia’s Institutional Animal Care and Use Committee in accordance with the Animal Welfare Act of the NIH. The human surfactant protein (SP)-C driven hemagglutinin (HA)-tagged HO-1-FL and C-terminal 53 amino acid Nuc-HO-1-TR cDNAs were independently engineered (Fig. 1a,b). Transgenic mice were generated using standard procedures of microinjection by the Transgenic Mouse Core Facility of the Children’s Hospital of Philadelphia [24].

### Hyperoxic Exposure and Recovery

Neonatal pups were randomly assigned to room air (normoxia) or 95% oxygen (hyperoxia). Exposure to hyperoxia was conducted for 72 hours in a chamber (BioSpherix, Redfield, NY), which allows for continuous monitoring and regulation of oxygen and carbon dioxide. Dams were switched every 24 h between normoxia and hyperoxia. Some mice were allowed to recover in room air to adulthood (8 months of age).

### Lung Tissue Collection

Mice were anesthetized with an intraperitoneal injection of ketamine hydrochloride (100 mg/kg) and xylazine hydrochloride (10 mg/kg). After the pulmonary artery was perfused with phosphate buffered saline (PBS), the right lung was excised and snap-frozen with liquid nitrogen for protein analysis. The left lung was inflated and fixed with 10% neutral-buffered formalin (HT5014, Sigma-Aldrich, St Louis, MO) for 24 hours. Lung tissue was paraffin-embedded and 5-µm thick sections were mounted on glass slides.

### HO Activity Assay

Carbon monoxide production was measured as a marker of HO activity using gas chromatography as previously described [25].

### Radial Alveolar Counts (RAC) and Alveolar Wall Thickness

Alveolarization was quantified by RAC, as described [26], [27]. The alveolar wall thickness was measured. One lung section from 5 separate animals per study group was viewed at ×20 magnification under horizontal lines. Thickness of each septum was measured parallel to the intersecting line utilizing ImageJ software (National Institutes of Health, Bethesda, MD).

### Detection of Protein Carbonylation

The detection of protein carbonylation was performed using a Protein Oxidation Detection Kit (Millipore, Billerica, MA), according to the manufacturer’s instructions.

### DNA Laddering Assay

Genomic DNA was isolated from frozen lung tissue using the BDtract genomic DNA isolation kit (Maxim Biotech, San Francisco, CA). Fragmented DNA was ligated to adaptor DNA fragments and subjected to PCR for amplification of cleaved genomic DNA according to the manufacturer’s instructions (PCR kit for DNA ladder assay, Maxim Biotech, San Francisco, CA). Samples were run on 2% agarose-ethidium bromide gel and visualized using a UV imager.

### Evaluation of Protein Levels in Lung Homogenates

Western analysis was performed to evaluate protein levels, as described [28]. The antibodies were as follows: anti-HA (clone 16B12, Covance, Richmond, CA), anti-HO-1 (SPA-896, Enzo Life Science, Plymouth Meeting, PA), anti-PARP (Cell Signaling Technology, Danvers, MA), anti-PAR (10H, Enzo Life Sciences International, Plymouth Meeting, PA), anti-PAR glycohydrolase (PARG) (Abgent, San Diego, CA), anti-lamin B (sc-6216, Santa Cruz Biochemistry, Santa Cruz, CA), and anti-calnexin (SPA-860, Stressgen, Victoria, BC, Canada), anti-phospho-p44/42 mitogen-activated protein kinase (MAPK) (extracellular signal-regulated kinase (ERK) 1/2) (Cell Signaling Technology, Danvers, MA), anti-ERK1 (Santa Cruz Biochemistry, Santa Cruz, CA), and anti- epidermal growth factor receptor (EGFR) (Cell Signaling Technology, Danvers, MA). Prior to western analysis of PAR, PARG enzyme (Trevigen, Gaithersburg, MD) was added to lung homogenate of mice and incubated at 37°C for 1 h.

### Immunohistochemistry

Paraffin-embedded tissue sections were processed for indirect immunofluorescence staining or a modified method using biotin amplification and a commercial kit (Tyramide Signal Amplification System, PerkinElmer, Waltham, MA). Sections were incubated overnight at 4°C with anti-HA (clone 16B12; Covance, Richmond CA), biotinylated anti- proliferating cell nuclear antigen (PCNA) (PC10: ab29, Abcam, Cambridge, UK), anti-pro-SP-C (AB3786, Millipore, Temecula, CA), anti-vimentin (Clone LN-6, Sigma-Aldrich, Saint Louis, MO), anti-alpha-smooth muscle actin (α-SMA; clone 1A4, Sigma-Aldrich, Saint Louis, MO), and anti-CD45 (clone 30-F11, BD Biosciences, San Diego, CA).

### Immunoprecipitation

Nuclear extracts from the WT or Nuc-HO-1-TR lung were prepared using the NE-PER Nuclear and Cytoplasmic Extraction Kit (Pierce, Rockford, IL). Immunoprecipitation was performed using anti-HA antibody conjugated to agarose beads (HA Tag IP/Co-IP kit, Pierce, Rockford IL). Briefly, tissue lysates were transferred to spin columns and anti-HA agarose beads were added followed by overnight incubation at 4°C. The columns were spun to remove the tissue lysate, and the beads were washed. The immunoprecipitated proteins were eluted and western blotting was performed.

### Assessment of Respiratory Mechanics

Eight-week-old mice were anesthetized, tracheostomized and connected via the endotracheal cannula to a flexiVent system (SCIREQ Inc., Montreal, Canada). Inspiratory capacity, resistance, compliance, elastance, tissue damping, and tissue elastance were calculated.

### Magnetic Resonance Imaging (MRI)

MRI was performed on 4-week-old mice. MRI images were obtained with a 7 Tesla ClinScan animal scanner (Bruker, Ettlingen, Germany) running the Syngo acquisition software (Simens, Malvern, PA). The lung was outlined in all coronal planes with a computer-assisted free-outline technique on images and the volume was calculated with the Vitrea Enterprise SuiteTM software (Vital Image, Minnetonka, MN).

### Chemotactic Invasion of HO-1 Stably Infected HO-1 Null Mouse Embryonic Fibroblast (MEF) Cells

A 0.5% solution of low–melting point agarose (Invitrogen, Carlsbad, CA) was made by boiling with PBS. The solution was cooled to 40°C, and mixed with either PBS alone or epidermal growth factor (EGF) being in a solution of 0.09 µg/100 µl. Ten-microliter spots of agarose containing EGF were spotted onto the slides and allowed to cool and 3×10^6^ cells were seeded onto the plates containing the slides in the presence of media with 10% fetal calf serum (FCS) and allowed to adhere for 4 h. Cells were transferred into cell culture media with 0.1% FCS and incubated for 7 hours at 37°C. The slides were then viewed under confocal microscope.

### Statistical Analysis

Values were the mean ± standard error of the mean (SEM) of separate experiments. For comparison between treatment groups, the null hypothesis that there is no difference between treatment means was tested by unpaired t-test for two groups. A p value <0.05 was considered significant.

## Results

### Expression and Localization of HA-tagged HO-1 in the Lung

Three transgenic lines overexpressing HO-1-FL(H), HO-1-FL(L), or Nuc-HO-1-TR were further characterized (Fig. 1c). Fluorescence microscopy verified cytoplasmic and nuclear HA staining of alveolar epithelial cells in the HO-1-FL and Nuc-HO-1-TR, respectively (Fig. 1d).

### Determination of the Enzymatic Activity of Lung HO-1

Total HO activity in HO-1-FL(H) was 4 times higher than that in wild-type (WT). HO activity was no different in the Nuc-HO-1-TR and HO-1-FL(L) compared with WT despite enhanced (2 fold increased) HO-1 protein (Fig. 1e).

### Alveolar Development is Impaired after Hyperoxia and Recovery

**Figure 2:**
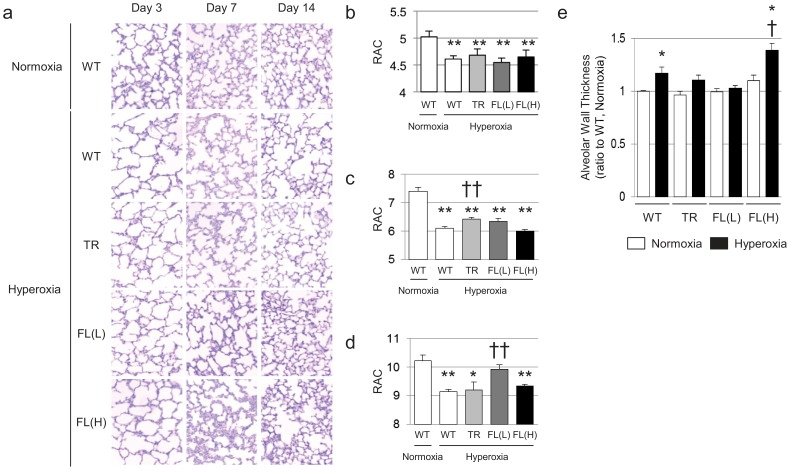
Morphological analysis of HO-1 trangenic mouse lungs after neonatal hyperoxic exposure and recovery in room air. (**a**) Hematoxylin and eosin (H&E) stained histological sections from animals on days 3, 7, and 14. (**b–d**) RAC on Days 3, 7, and 14. Each group had a minimum of 6 samples, and data are the mean ± SEM. *, p<0.05 vs normoxia **, p<0.01 vs normoxia; ††, p<0.01 vs WT/hyperoxia. (**e**) Alveolar wall thickness on day 7. Values from WT control (WT, normoxia) were set at 1 to calculate the relative values in other experimental groups. Each group had a minimum of 5 samples, and data are the mean ± SEM. *, p<0.05 vs normoxia; †, p<0.05 vs WT/hyperoxia.

In air, the WT developed well-organized terminal airways. In contrast, exposure of WT newborn mice to 3-day hyperoxia impaired alveolar development, resulting in alveolar simplification (Fig. 2a) and RAC were significantly reduced (Fig. 2b). Nuc-HO-1-TR, HO-1-FL(L), and HO-1-FL(H) showed reduced RAC after 3 days of hyperoxia (Fig. 2b). When WT were exposed to hyperoxia for 3 days then allowed to recover in room air for 11 days, they still had significantly decreased RAC, compared to their normoxic counterparts (Fig. 2b–d), whereas, despite worsened RAC after acute hyperoxia, the HO-1-FL(L) had significantly improved RAC after room air recovery compared with the similarly exposed WT (Fig. 2d). In contrast, neither the Nuc-HO-1-TR or the HO-1-FL(H) showed improved RAC after an 11-day recovery.

### HO-1-FL(H) Exhibit Thickened Alveolar Walls with Hypercellularity when Recovering from Hyperoxic Injury

Hyperoxia-exposed WT exhibited a decrease in alveolar wall thickness at 3 days of age (not shown) and a subsequent increase at 7 days of age compared with the normoxia exposed controls (Fig. 2e). Similarly, HO-1-FL(L) demonstrated reduced alveolar wall thickness after hyperoxia (not shown), but did not exhibit increased wall thickness during recovery (Fig. 2e). In contrast, HO-1-FL(H) had persistently increased alveolar wall thickness with hypercellularity during hyperoxia and recovery (Fig. 2a,e).

### HO-1-FL(H) Demonstrate Increased Lung Cell Proliferation and Decreased Apoptosis after Hyperoxic Exposure

**Figure 3:**
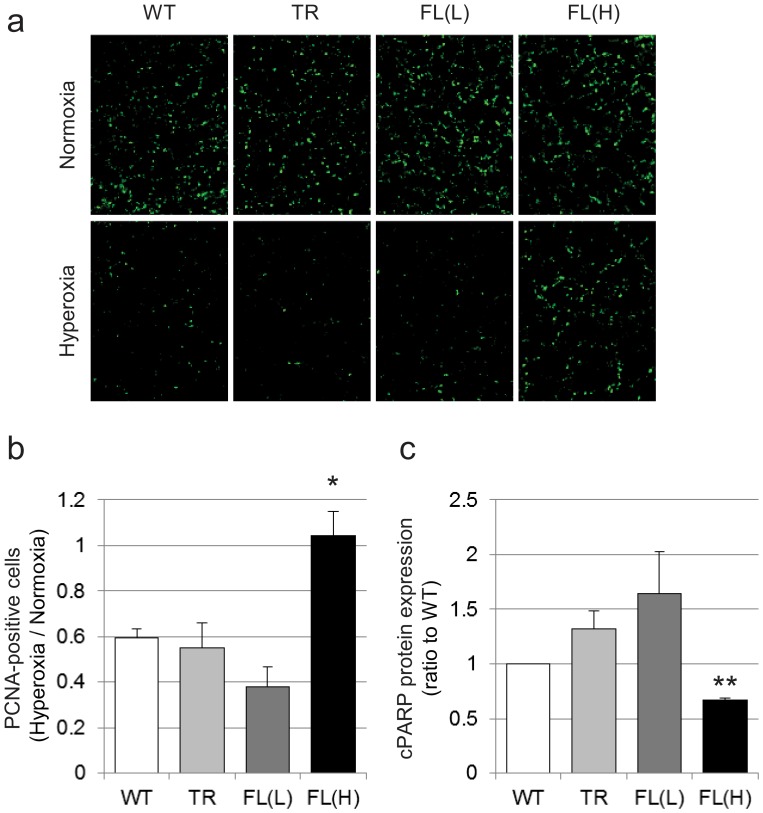
Evaluation of cell proliferation and apoptosis in the HO-1 transgenics exposed to neonatal hyperoxia. (a) PCNA (green) immunostaining on Day 3. (b) Quantification of PCNA immunopositive cells. Five high powered fields were counted in each lung. Each group had 3 samples, and values are expressed as a ratio to air and are the mean ± SEM. *, p<0.05 vs WT. (c) Quantification of cleaved PARP protein levels in HO-1 transgenic mice exposed to 3-day hyperoxia on Day 7. Values are the mean ± SEM of 3 determinations in each group. **, p<0.01 vs WT.

To understand whether increased alveolar wall thickness in HO-1-FL(H) was due to altered cellular proliferation and/or apoptosis, immunohistochemical staining for PCNA, a general marker of cell proliferation, and western blot analysis for cleaved PARP, a marker of apoptosis [29], were used. In WT, Nuc-HO-1-TR, and HO-1-FL(L), after hyperoxic exposure, the number of PCNA-positive cells was decreased. However, this was not the case in the HO-1-FL(H) (Fig. 3a,b). Furthermore, HO-1-FL(H) had less apoptosis after exposure to 3-day hyperoxia compared to WT (Fig. 3c).

### HO-1-FL(L) have Decreased Evidence of Oxidative Injury after Hyperoxia and Recovery

**Figure 4:**
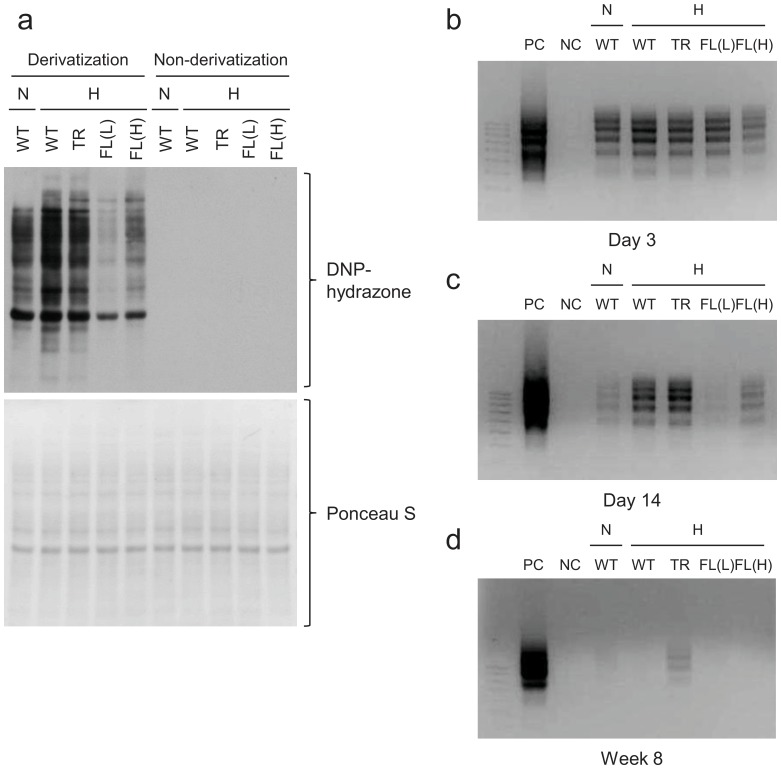
Oxidative stress status of protein and DNA after hyperoxic exposure. (**a**) Evaluation of dinitrophenol (DNP) immunoreactive signal in the HO-1 transgenic lungs after neonatal hyperoxic exposure. Non-derivatized proteins were loaded as negative controls. Membranes were stained with Ponceau S as a loading control. Representative image of DNA laddering in lung homogenates from 3 day old (**b**), 14 days old (**c**), and 8 week old (**d**) HO-1 transgenics exposed to hyperoxia as neonates.

Since HO-1 is an antioxidant molecule [30], we verified whether overexpression of HO-1 modulated markers of oxidative stress. As expected, increased protein carbonylation was observed in the WT after 3-day hyperoxia (Fig. 4a, lane 2), whereas HO-1-FL(L) exhibited a marked decrease in lung oxidized proteins after hyperoxic exposure compared with WT (Fig. 4a, lanes 2–5).

### Adult Nuc-HO-1-TR Exposed to Hyperoxia as Neonates have Persistent DNA Damage

A known consequence of hyperoxia is DNA oxidative damage [31]. Whereas neonatal mice exposed to hyperoxia had increased lung DNA fragmentation, which persisted for up to 2 weeks (Fig. 4b,c). This resolved by adulthood in all groups (Fig. 4d) except for the Nuc-HO-1-TR, which had increased lung DNA fragmentation at 14 days of age compared with other transgenic lines and persistence into adulthood (Fig. 4c,d).

### HO-1-FL(H) Exposed to Hyperoxia as Neonates have Thickened Alveolar Walls and Abnormal Alveolar Type II Cell Proliferation

**Figure 5:**
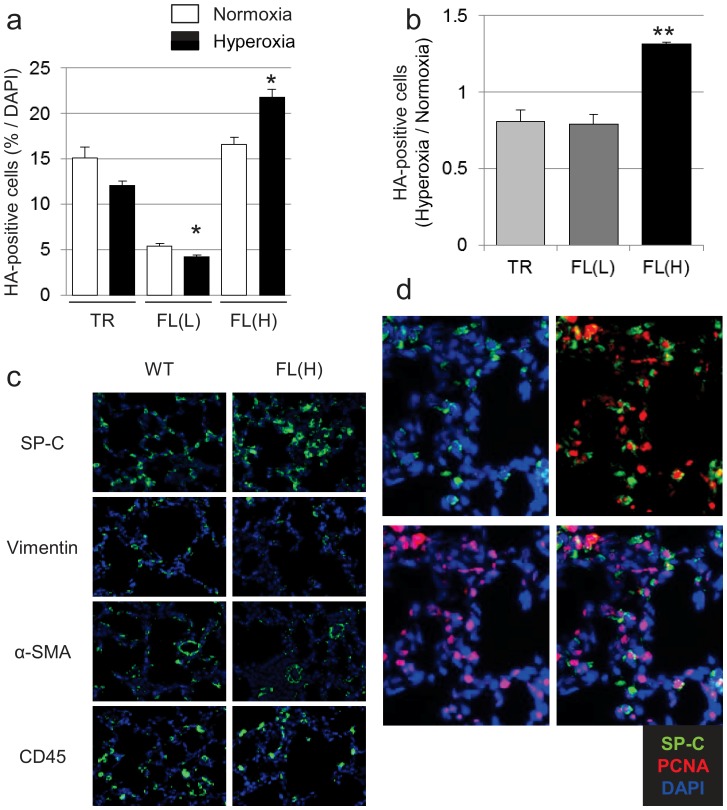
Evaluation of HO-1 overexpressing epithelial cells in HO-1 transgenic mice. (**a**) Quantification of HA immunopositive cells corrected for total DAPI-positive nuclei. Values are the mean ± SEM of 3 separate determinations. *, p<0.05 vs normoxia. (**b**) Quantification of HA immunopositive cells. as a ratio to air exposed controls. Values are the mean ± SEM of 3 separate determinations. **, p<0.01 vs TR, FL(L). (**c**) Immunostaining with cell-specific markers SP-C, vimentin, α-SMA, and CD45 (green), nuclei are stained with DAPI (blue). (**d**) Coimmunostaining of SP-C (green), PCNA (red) and DAPI (blue) in the HO-1-FL(H) transgenics exposed to hyperoxia as neonates.

The increased cell proliferation and decreased apoptosis in the lung after hyperoxia could explain the hypercellularity and thickened alveolar walls seen in HO-1-FL(H). In fact, the HO-1-FL(H) had significantly increased HA-positive cells in the lung after hyperoxia compared to other groups (Fig. 5a,b) and regions of thickened alveolar walls and hypercellularity compared with WT and the other 2 transgenic lines (Fig. 2a,e). Using cell specific antibodies, we verified that there was no increase in fibroblasts, myofibroblasts, and inflammatory cells in HO-1-FL(H). However, increased numbers of alveolar type II cells were identified in the thickened alveolar walls of the HO-1-FL(H) (Fig. 5c) and co-localization of anti-pro-SP-C and anti-PCNA immunofluorescent staining was observed (Fig. 5d). In addition, the accumulation of type II cells was not diffuse but focal.

### Nuc-HO-1-TR show Increased PARP and Decreased PAR Hydrolysis during Recovery from Hyperoxia

**Figure 6:**
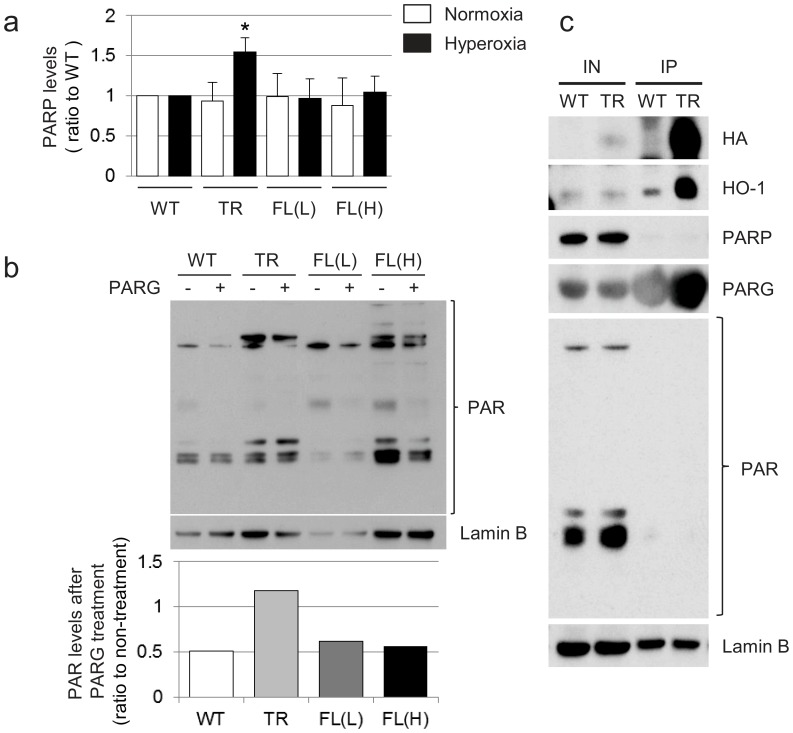
Assessment of lung PARP and PAR hydrolysis in 2 week old HO-1 transgenic mice exposed to hyperoxia as neonates. (**a**) Values are the mean of 3 densitometric measurements in each group. *, p<0.05 vs WT. (**b**) Quantification of PAR immune signal after incubation with PARG enzyme in the 14 day old HO-1-transgenic mice. Lamin B is shown as a loading control. (**c**) Immunoprecipitation of Nuc-HO-1-TR with PAR-related proteins. Representative PARG signal after immunoprecipitation with HA-tagged Nuc-HO-1-TR. Lamin B is shown as a loading control. IN, input; IP, immunoprecipitation.

An important mediator of DNA repair is PARP. We have previously shown that PARP binds to nuclear HO-1 *in vitro* [23]. Therefore, the relationship between Nuc-HO-1-TR and PAR-related proteins was investigated. Total PARP protein levels were increased in Nuc-HO-1-TR during recovery from hyperoxia compared with WT (Fig. 6a) and PARG did not hydrolyze PAR in Nuc-HO-1-TR (Fig. 6b) in contrast to WT, HO-1-FL(L), and HO-1-FL(H). Furthermore, PARG was pulled down with HA-tagged nuclear HO-1, suggesting that nuclear HO-1 also binds PARG (Fig. 6c). This binding may limit PARG-mediated PAR hydrolysis leading to its accumulation.

### Nuc-HO-1-TR and HO-1-FL(H) Exposed to Hyperoxia as Neonates have Diminished Pulmonary Function in Adulthood

**Figure 7:**
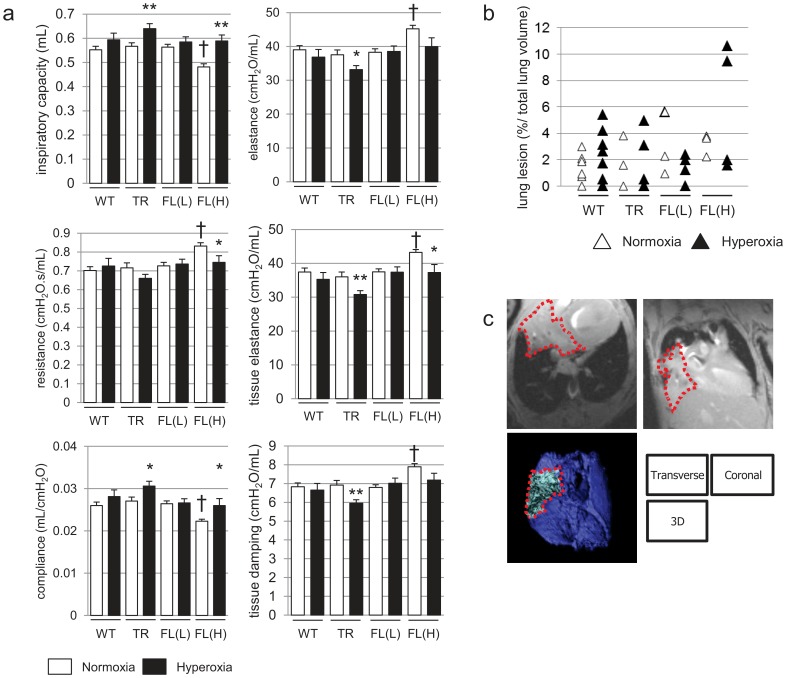
Pulmonary function and MRI in adulthood. (**a**) Respiratory mechanics in 8 week old HO-1 transgenic mice exposed to hyperoxia as neonates. Inspiratory capacity, resistance, compliance, elastance, tissue elastance, and tissue damping, were measured. Values are the mean ± SEM of 3 separate determinations in each group. *, p<0.05; ** vs normoxia; p<0.01 vs normoxia; †, p<0.01 vs WT. (**b, c**) Lung MRI in 4 day old HO-1 transgenic mice exposed to hyperoxia as neonates. (**b**) Percent ratio of lung density to total lung volume calculated using the Vitrea software after outlining the lung with a computer-assisted free-outline technique. Each value is shown as an empty (normoxia) or filled (hyperoxia) triangle. There are no statistical differences among groups. (**c**) Representative transverse, coronal, and three-dimensional reconstruction images of HO-1-FL(H) lungs exposed to 3-day hyperoxia as neonates. Dotted line outline the lung density.

Indices of pulmonary function were assessed in the mice exposed to hyperoxia as neonates at 8 weeks. In HO-1-FL(H) exposed to air, inspiratory capacity and compliance were decreased, whereas resistance, elastance, tissue elastance, and tissue damping were increased compared to WT, and exposure to hyperoxia adversely affected these parameters (Fig. 7a). Consistent with the improved RAC seen at 2 weeks in HO-1-FL(L), neonatal hyperoxic exposure did not change pulmonary function in this group. Nuc-HO-1-TR had increased compliance and decreased elastance compared to WT (Fig. 7a).

### HO-1-FL(H) have Pulmonary Densities on MRI

Although exposure to hyperoxia as neonates resulted in increased total lung volume as quantified from MRI digital stacks (not shown), there were no statistical differences in total lung volume between each group. Nevertheless, the 4 week old HO-1-FL(H) exposed to 3-day hyperoxia as neonates had increased pulmonary densities on MRI (Fig. 7bc).

### HO-1-FL(H) Exposed to Hyperoxia as Neonates Show Evidence of Abnormal Cell Proliferation as Adults

**Figure 8:**
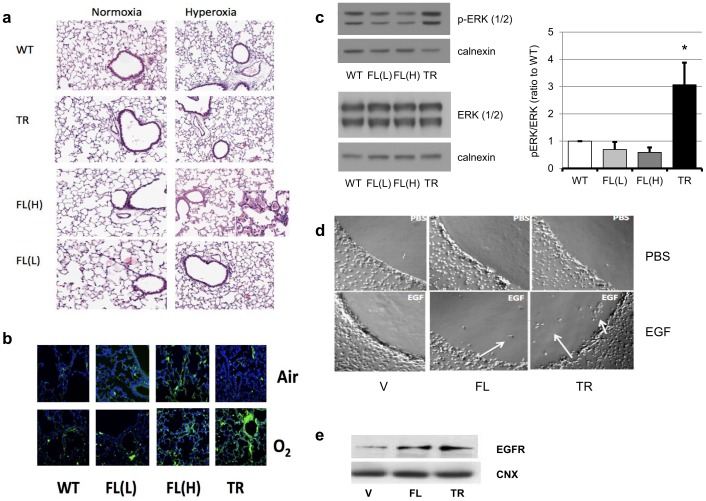
Cell proliferation and tumorigenesis during repair from hyperoxia as adults. (**a**) Representative H&E staining of lung slices from 8 week old HO-1 transgenic mice exposed to hyperoxia as a neonates. Inset: 40×magnification showing abnormal multinucleated intra-alveolar cells. (**b**) Representative p-ERK immunosignal in lung slices from 8 week old HO-1 transgenic mice exposed to hyperoxia as neonates. P-ERK is shown in green and DAPI in blue. (**c**) Left-upper panel: representative of 3 western analyses showing total ERK signal in lung homogenates from 8 week old HO-1 transgenic mice exposed to hyperoxia as neonates. Left-lower panel: representative of 3 western analyses showing p-ERK signal in lung homogenates from the same animals. Calnexin serves as the loading control. Right panel: Densitometric evaluation of pERK/ERK ratio from the blots in the left panel. Values are the mean ± SEM of 3 densitometric measurements in each group. *, p<0.05 vs WT, FL(L), and FL(H). (**d**) Representative migration of HO-1 infected HO-1 null MEF towards an agarose spot containing EGF. Arrows: migrated cells. (**f**) Representative of 2 western blots for EGFR immunosignal in HO-1 infected HO-1 null MEF cells. Calnexin is the loading control.

To understand what contributed to the pulmonary densities seen on MRI in HO-1-FL(H), we re-assessed lung histology in all groups at 8 weeks. Only the lungs of HO-1-FL(H) exposed to hyperoxia as neonates showed abnormal multinucleated intra-alveolar cells without evidence of inflammation or fibrosis (Fig. 8a). Since an association between HO-1 abundance and/or nuclear localization and tumor cell proliferation has been implied [21], [32], [33], we assessed the lung tissues and homogenates in all groups for markers of tumorigenesis [34] at 8 weeks. Neither EGFR nor phosphorylated (p)-ERK immunoreactivity was elevated in WT, HO-1-FL(L), or HO-1-FL(H), but p-ERK was increased in Nuc-HO-1-TR. To further test whether localization or abundance of HO-1 promotes tumorigenesis during repair from hyperoxia, migration of HO-1 null MEF cells stably infected with HO-1-FL or Nuc-HO-1-TR cDNAs towards a solution of low–melting point agarose containing PBS with or without EGF was documented. Both HO-1-FL and Nuc-HO-1-TR MEF cells exhibited migration towards agarose with EGF although this was more evident in Nuc-HO-1-TR (Fig. 8d). In corroboration, Nuc-HO-1-TR cell had increased abundance of the EGFR (Fig. 8e). These data suggest that nuclear HO-1 promotes tumor-like behavior *in vitro*.

## Discussion

Although hyperoxia is not found in nature, it is common in the clinical setting and adaptive responses exist to this challenge. Induction of HO is observed in adult rodents exposed to hyperoxia and is thought to be cytoprotective [8]. However, lung HO-1 is not induced in similarly exposed neonatal mice [9]. Furthermore, neonatal mice have increased nuclear localization of lung HO-1 in hyperoxia and adults do not [23]. We have previously demonstrated that despite beneficial effects of HO-1 at low levels of expression, there was a reversal of cytoprotection with increased HO-1 expression *in vitro* [13], indicating a beneficial threshold of HO-1 overexpression. Ours is the first report demonstrating that the degree of HO-1 overexpression and intracellular localization alters its cytoprotective abilities *in vivo*. We successfully generated two HO-1-FL transgenic mouse lines expressing HO-1 protein in the lung at low and high levels. In addition, since localization of HO-1 in the nucleus is associated with various cytoprotective effects [22], lung-specific transgenic mice expressing HO-1 protein in the nucleus were also generated. To simulate the clinical circumstance, mice were exposed to hyperoxia for 3 days then recovered in air. This model allows for an assessment of acute injury as well as repair [35].

Despite no significant increase in HO activity and similar levels of arrested alveolarization, alveolar wall thinning, and decreased alveolar cell proliferation than WT after 3 days of hyperoxia, HO-1-FL(L) recovered completely from lung injury induced by neonatal hyperoxia by 14 days. This may be due to mitigation of oxidative stress during the recovery period. The lack of long-term adverse effects on lung structure and pulmonary mechanics suggests that moderate overexpression of HO-1 protein in pulmonary epithelial cells may play a critical role in the resolution of hyperoxia-induced acute lung injury by reducing oxidative stress in type II epithelial cells that are critical targets for hyperoxia-mediated impairment and recovery of postnatal lung development [36]. Interestingly HO activity was not different than WT in this model suggesting that a modelrate change in HO-1 protein, even without activity is sufficient to provide protection. We have previously shown that HO-1 protein even if not catalytically active [37] can alter transcription factor activation and also still provide protection against oxidative stress *in vivo* [13]. This model suggests that the same is true *in vivo*. It could be that the lack of difference in HO activity is due to the fact that the sample represents the whole lung homogenate and that the activity resides only in the type II cells. This remains to be determined.

If moderate overexpression of HO-1 is protective, it seems counterintuitive that further overexpression of HO-1 would worsen hyperoxia-induced lung injury. In fact, this resulted in increased cell proliferation, decreased apoptosis, focal type II cell accumulation, and thickened alveolar walls, contributing to long-term physiological changes in pulmonary function and increased pulmonary densities. The expression of HO-1 is often enhanced in cancer cells, as demonstrated in prostate, brain, pancreatic, and lung cancers as well as several other tissues [38]–[48]. HO-1 is highly upregulated in rapidly proliferating cells such as in the epithelium within wounded skin or psoriatic lesions. In contrast, HO-1 inhibition reduces the viability of colon carcinoma, acute myeloid leukemia, and hormone-refractory prostate cancer [39], [49], [50]. It could be that the HO-1-FL(H) with alveolar wall thickness and hypercellularity are prone to malignant transformation. In fact, we observed abnormal cells within the alveoli of HO-1-FL(H) and enhanced migration towards EGF *in vitro* after stable transfection with HO-1-FL cDNA. The abnormal pulmonary densities were predominantly seen after hyperoxic exposure, suggesting that the pro-proliferative and perhaps tumorigenic effects of high HO-1 overexpression were facilitated by hyperoxia. Interestingly, these cells did not bear the typical signature of other lung cancers. It remains to be determined if this would evolve over time. Nevertheless, the maladaptive accumulation of Type II cells also suggests that there is a derangement of the normal repair process which results in Type II to Type I transdifferentiation to maintain proper lung architecture and that this would lead to a persistently abnormal lung architecture.

We were intrigued that we did not see pulmonary densities in the Nuc-HO-1-TR mice because nuclear localization of HO-1 has been associated with malignant transformation and metastasis in several other models [19]–[21]. Although there were no statistically significant differences in the number of pulmonary densities seen on MRI in Nuc-HO-1-TR, these animals had increased lung p-ERK signaling and MEF stably transfected with Nuc-HO-1-TR cDNA had increased EGFR expression and increased migration towards EGF, further suggesting a tumorigenic potential. Perhaps, with a longer recovery period, the animals would develop pulmonary foci or abnormal cells.

In the case of the Nuc-HO-1-TR, nuclear HO-1 was increased on a background of endogenous cytoplasmic HO-1. Nevertheless, this model allowed us to evaluate the effects of enhanced nuclear HO-1 on hyperoxic lung injury and was more relevant to the *in vivo* situation in neonates. We have recently shown that PARP is one of the candidate binding partners of HO-1 in the nucleus *in vitro* [23]. Activation of PARP is a cellular response to DNA single strand breaks. Once PARP detects DNA single strand breaks, it binds to the DNA and begins to synthesize a poly PAR chain as a signal for other DNA-repair enzymes, such as X-ray cross-complementing gene 1 [51] and aprataxin polynucleotide kinase phosphatase-like factor [52]. Thereafter, the PAR chains are degraded via PARG [53]. These events facilitate DNA repair. However, the accumulation of PAR could also play a role in PARP-dependent cell death [54], [55]. We demonstrated that the interaction of nuclear HO-1 and PARG proteins altered the activity of PARG. We suspect that this led to the persistent DNA damage and subsequent emphysematous phenotype seen on histology and with pulmonary function testing in Nuc-HO-1-TR. This demonstrates that the nuclear HO-1 mediated inhibition of DNA repair in type II cells hinders repair from hyperoxic injury.

In summary, using lung-specific HO-1 transgenic mice, we have demonstrated that there is a beneficial threshold of HO-1 protein overexpression in the lung *in vivo*. Although moderate to low levels of HO-1 expression are beneficial due to inhibition of oxidative damage, high overexpression of HO-1 is harmful due to abnormal cell proliferation and decreased apoptosis, which have both short term and long term-consequences on lung function and structure. Also, overexpression of nuclear HO-1 inhibits repair from hyperoxic lung injury by inhibiting DNA repair, which may predispose the lung to later malignant transformation. A clearer understanding of the nuances of HO-1 cytoprotective effects is important for developing effective therapeutic strategies to prevent lung oxidative injury and tumorigenesis.
